# Food and body-related attentional biases in children and adolescents with eating disorder symptoms, overweight and obesity: a systematic review

**DOI:** 10.1186/s40337-025-01459-9

**Published:** 2025-12-10

**Authors:** Maarit Pelzer, Timo Brockmeyer, Brunna Tuschen-Caffier, Jessica Werthmann

**Affiliations:** 1 Department of Clinical Psychology and Psychotherapy, Institute of Psychology, Albert-Ludwigs-University Freiburg, Freiburg im Breisgau, Germany; 2 Institute of Psychology, Department of Psychology, Clinical Psychology and Translational Psychotherapy, University of Münster, Münster, Germany

**Keywords:** Childhood and adolescence, Eating disorders, Overweight/obesity, Attention bias, Food-related stimuli, Body-related stimuli, Systematic review

## Abstract

**Introduction:**

Disordered eating behaviors (DEBs), including restrictive eating, binge eating and purging, are associated with mental health problems and an increased risk of eating disorders (EDs), which often occur in adolescence and can have serious health consequences. In addition, the increasing rates of overweight and obesity among children and adolescents raise concerns about their associated physical and mental health risks. Attentional biases (ABs) to food- and body-related cues have been proposed as cognitive mechanisms that contribute to the development and maintenance of EDs and are also discussed in the etiology of overweight and obesity. While theoretical models suggest that ABs may contribute to the maintenance of EDs, DEBs, and obesity, empirical evidence in young populations is still limited.

**Method:**

This systematic review (PROSPERO: CRD42023399292) examined literature from PsycINFO, PubMed, and Scopus on ABs to food- and body-related stimuli in children and adolescents with overweight, obesity, or ED symptoms, compared to healthy comparisons. A total of 30 peer-reviewed studies published in English since 2003 were included.

**Results:**

The evidence on AB for food in young people with overweight and obesity remains inconclusive, and studies provide conflicting results. Similarly, studies in adolescents with AN show heightened attention to low-calorie foods and inconsistent attentional patterns toward high-calorie foods, indicating a complex and heterogeneous picture. Evidence on AB for food in young people with BED is scarce, with one study reporting an attentional bias towards food. For LOC eating, findings were mixed and less conclusive. This suggests that body-related AB may serve as a relevant marker for the psychopathology of EDs, particularly in AN.

**Conclusion:**

This review underscores the role of ABs in EDs and overweight/obesity and highlights methodological inconsistencies as well as research gaps, particularly in samples beyond AN and overweight/obesity. Future studies should therefore employ standardized methods, diverse samples, and developmental perspectives to improve understanding of AB in the etiology of these pathologies and inform targeted interventions for at-risk youth.

**Supplementary Information:**

The online version contains supplementary material available at 10.1186/s40337-025-01459-9.

## Abstract (plain language summary)

Eating disorders (EDs) and unhealthy eating behaviors can seriously affect young people’s mental and physical health. In addition, the increasing rates of overweight and obesity among adolescents raise concerns about their associated physical and mental health risks. One possible factor influencing eating behavior and body perception is attentional bias (AB) – the tendency to selectively filter certain information, such as food or body-related cues. This review systematically examines studies on AB in young people with EDs, overweight, or obesity. Findings on food-related ABs were inconsistent, with some studies showing increased attention to food in young people with obesity/overweight or EDs compared to comparison participants, while others found no clear differences or even decreased attention to food. In contrast, research on body-related ABs in anorexia nervosa (AN) was more consistent, showing a stronger focus on thin bodies and self-perceived unattractive body areas in young people with AN compared to comparison participants. These findings suggest body-related ABs may play a role in AN, yet, overall, research remains limited and inconsistent. More studies using reliable methods are needed to clarify similarities and differences in ABs across different eating behaviors and to provide a basis for improving prevention and strategies for at-risk children and adolescents.

**Supplementary Information:**

The online version contains supplementary material available at 10.1186/s40337-025-01459-9.

## Introduction

### Disordered eating behavior, eating disorder and prevalence in youth

Eating-related problems, including loss of control eating and emotional eating or unhealthy weight control behaviors such as purging, food restriction, dieting, are described as disordered eating behaviors (DEBs) by the American Psychiatric Association [1] and the American Dietetic Association [2]. Longitudinal studies link DEBs to psychological issues in both children and adults, and these behaviors can lead to stress, anxiety, and abnormal weight fluctuations, impacting daily life [3, 4].

Chronic unhealthy or pathological eating habits may be diagnosed as eating disorders (EDs; [5]) typically emerging in mid to late adolescence with a peak of onset between 15 and 25 years [6, 7]. They are associated with early mortality, comorbid health issues and functional impairment [6, 8, 9]. A recent narrative review reported that in Western settings overall, 5.5–17.9% of young women and 0.6–2.4% of young men have experienced a Diagnostic and Statistical Manual, 5th edition (DSM-5; [1]) ED by early adulthood [10].

Another pressing problem related to eating behavior is the high prevalence of obesity and overweight in young people, posing significant psychological and physical health risks, such as cardiovascular disease (mainly heart disease and stroke), type 2 diabetes and EDs [11]. The World Health Organization (WHO) estimates that 2.8 million people die each year due to the adverse consequences of overweight and obesity [12]. In 2022, an estimated 37 million children under the age of 5 years and over 390 million children and adolescents aged 5–19 years were overweight [13]. Thus, efficient interventions to target unhealthy and problematic eating behavior in young people are urgently needed. Studying mechanisms that maintain unhealthy and problematic eating behavior may offer an avenue for treatment. Attentional biases (ABs) could be one such potential mechanism. ABs are early processing tendencies to overly focus on disorder-, motivation- or mood-related information, which can disrupt balanced processing and can lead to selective, biased information intake [e.g., 14]. This is particularly relevant because the processing of information (i.e., attention processing) subsequently influences our emotions and our behavior. Hence, ABs play a prominent role for behavior disorders, like EDs, according to cognitive-behavioral theories [15, 16]. Indeed, food-related attention processing (e.g., a tendency to allocate attention faster or longer on food cues in the environment) have been implicated in craving, overeating and obesity and also with EDs and restrained eating [17–20].

In addition to food-related biases, body-related biases (e.g., a stronger focus of attention on slim bodies in the environment) may further contribute to the development and/or maintenance of EDs. EDs are characterized by dysfunctional cognitions and maladaptive schemas about weight and body shape [21, 22]. The activation of these cognitions and schemas can bias attention for ED-related stimuli such as body-related cues [22] and may contribute to body dissatisfaction, body-related avoidance, and body-checking behaviors [21, 23]. Body dissatisfaction, body-related avoidance, and body-checking behaviors are also common in people with obesity [24, 25], suggesting that body-related ABs may underlie body-related problems in both EDs and obesity. While theoretical models suggest that food- and body-related ABs may contribute to the development and maintenance of EDs, overweight and obesity, empirical evidence in young populations is still limited.

In this article, we aim to review the results of studies measuring AB toward food- and/or body-related stimuli in young people and the association of these biases with EDs and overweight/obesity. We consider studies with direct (e.g., eye-tracking, electroencephalography) and indirect measures (e.g., Stroop or visual probe tasks) of attention.

### The role of ABs for food in overeating and obesity

Food is essential for survival and especially high-calorie food cues are potent in quickly capturing attention [20, 26]. In today’s “obesogenic” food environment, palatable, high-calorie, cheap and convenient foods are constantly available and promoted aggressively, contributing to the rise of (childhood) obesity [27] and making healthy weight maintenance challenging [28, 29]. In this environment, an AB for high-calorie food may trigger consumption [30]. This has contributed to (political) discussions, e.g., in Germany, to restrict advertising for sweets and unhealthy foods [31].

It has been proposed that an AB for food develops through learning [32], with Robinson and Berridge’s [33] incentive sensitization model for addiction adapted to explain how an AB contributes to overeating and obesity [34]. According to this model, food cues are perceived as signal of imminent consumption, which is experienced as rewarding [35]. Over time, these food cues then gain incentive properties and become more and more salient during a conditioning process of repeated signaling and subsequent consumption.

Indeed, food-related ABs have been associated with cravings, increased intake, and weight gain in both children and adults across numerous studies [34, 36–43]. However, a recent meta-analysis limited to adult populations found no clear evidence of an increased AB for food in adults with overweight or obesity versus adults with healthy weight [43]. For instance, the results tend to suggest that food-related ABs are sensitive to changes in the motivational value of food but are not related to individual differences in body weight [43]. In addition, a major draw-back of the existing studies in adults is the great diversity in methodology (e.g., paradigms, stimulus categories and content) which impedes a general conclusion on the obtained results. In contrast to studies with adults, far less studies have focused on the topic of an AB for food, obesity and eating behavior in young people.

### The role of ABs for food in disordered eating behavior

In the context of EDs, theoretical models propose that food cues are associated with fear of gaining weight or losing control overeating, i.e. anxiety- or worry-related cognitions about eating and avoidance tendencies [15, 44]. In addition, it has been suggested that reactivity to food cues may be suppressed by cognitive control processes, or that alternative reward systems may have been established, in which not eating or eating very little is perceived as rewarding [45, 46]. Yet, similar to the incentive sensitization theory in obesity, food cues are also associated with craving and (binge-)eating [see e.g., 44], thus with reward-related cognitions and with approach tendencies, EDs, at least in binge eating disorder (BED), bulimia nervosa (BN) and anorexia nervosa (AN) with binge/purge symptoms.

Reviews on food-related ABs in EDs suggest that individuals with EDs and people with restrained eating exhibit a stronger AB towards food cues with small to medium effect sizes [17, 18, 48]. However, many of the early studies of AB for food in adults with EDs relied on the Stroop task, which cannot inform on the specific attention mechanisms (such as early or late attention processes or approach versus avoidance tendencies). More recent studies, using more refined measures which can distinguish between different attention mechanisms, reveal a less coherent picture (see e.g [49–51]). Overall, results are conflicting on whether individuals with EDs have an early attentional vigilance for food, try to avoid looking at food cues or are more distracted by food cues, when compared to healthy control participants [20]. Most studies on a food-related AB in ED samples have been conducted in adults and therefore little is known about the role of food-related ABs in young people.

In summary, reviews and meta-analyses on studies in adults have drawn the conclusion that obesity as well as ED symptomatology are associated with an AB for food. However, the findings are mixed and partly inconsistent, particularly regarding the exact role and nature of food-related ABs in EDs. Moreover, studies in children and adolescents are often not included in these reviews or not analyzed separately from adult samples. Consequently, a systematic overview of the current state of research specifically in children and adolescents is still lacking.

### The role of AB to body and shape-related stimuli in EDs, overweight and obesity

Shape concerns and fear of weight gain are key symptoms of individuals with EDs and have been associated with AB toward negatively valenced body- and shape-related stimuli in numerous studies (for a review, see [52, 53]). Several researchers argue that this dysfunctional attentional focus on problem-relevant stimuli is a cognitive correlate of EDs and it has been proposed that body-related AB are a possible risk factor for the development and maintenance of a distorted body image and body dissatisfaction [54]. Women and girls with ED symptoms are more likely to show dysfunctional and deficit-oriented gaze behavior, especially when looking at their own bodies (for an overview, see [54]). In contrast, healthy women tend to focus more on positive body parts [55]. Yet, note, that findings on body-related attention in healthy individuals are mixed as well: While some studies report balanced attention allocation between positive and negative body areas [56], others show that healthy women also (similar to findings in individuals with EDs) showed a deficit-oriented gaze behavior [57, 58]. These inconsistencies may be explained by varying levels of body dissatisfaction, with higher dissatisfaction leading to a stronger bias toward unattractive body areas [59]. However, Warschburger et al. [60] found that women with overweight who reported high body dissatisfaction focused more strongly on attractive regions of their own body, possibly as a self-esteem protection strategy.

Overall, studies comparing AB for liked and disliked body parts between adults with EDs (or with high body dissatisfaction) and healthy individuals (or with low body dissatisfaction) are inconsistent. Furthermore, given that higher body dissatisfaction is linked to ABs, and considering that body-related dissatisfaction is particularly common among youth [61] and continues to rise during adolescence [62, 63], it is important to systematically review the evidence on ABs related to body stimuli in children and adolescents.

### Definition and measurement of AB

Reviewing findings on ABs is complex because its operationalization varies by the attentional process measured, the assessment method and paradigm used, and the type of control stimuli selected. AB is often conceptualized as the preferential orientation of attention toward disorder-relevant stimuli or the sustained maintenance of attention on such stimuli – distinguishing between early, automatic processes and later, more controlled attentional allocation [64–66], and also encompasses processes such as avoidance of disorder-relevant stimuli, suppressing irrelevant stimuli, selecting salient cues, and deeper cognitive processing of relevant information.

AB can be assessed using indirect or direct methods. Indirect measures infer AB from differences in reaction time (RT) or accuracy to disorder-relevant versus neutral stimuli. Common paradigms include the Stroop, dot-probe, and visual search tasks. Less frequently used but conceptually relevant paradigms include the Attentional Blink (based on rapid serial visual presentation), which assesses attentional accuracy under high temporal load; the Attentional Response to Distal versus Proximal Emotional Information (ARDPEI) task, which captures attentional engagement and disengagement in response to personally salient stimuli; the Oddball Task, where participants respond to infrequent, salient targets (e.g., food) presented within a stream of neutral stimuli; and the Imbedded Words Task (IWT), which assesses AB via timed detection of salient versus neutral words.

Direct measures include eye-tracking (ET) and electroencephalography (EEG). ET provides continuous, spatially precise data on gaze behavior, allowing for fine-grained analyses of orienting and maintenance. EEG, particularly through event-related potentials (ERPs), offers high temporal resolution and captures both the timing and intensity of attention-related processing. Components such as the P3/P300 and late potentials are interpreted as markers of AB, reflecting increased perceptual allocation to stimuli. Many studies combine these approaches (e.g., RT-based paradigms, like the Oddball Task with EEG) to provide a more comprehensive assessment. Table 1 provides an overview about most commonly applied paradigms and measurement methods across the literature on AB in individuals with EDs or overweight/obesity.

**Table 1:** Overview about different methodologies to assess ABs

Methodology	Description	Direct vs. indirect	Attention Bias Index	Advantages	Disadvantages
Stroop task	Participants indicate the color of critical/non-critical words	Indirect	Differences in response time between biased (e.g., food-related words) and neutral words, indicating interference or slowed processing [137]	Simple to administer; highlights general attentional bias; historical significance in psychopathology research	Poor psychometric properties [138]; unclear direction of attention (approach vs. avoidance) [139]; no distinction of early vs. late attention [140]
Visual probe task	Two stimuli (critical and non-critical) shown side-by-side; probe replaces one stimulus	Indirect	Response latency indicates attention [141], early attention assessed by short stimulus times (100–500 ms); late attention by longer stimulus times (≥ 500 ms) [142, 143]	Distinguishes early vs. late attention; calculates direction of attention (approach vs. avoidance) [143]; widely applied	Poor reliability and internal consistency [138]; limited ecological validity; relies on inferred attention processes
Exogenous cueing task	Single stimulus (cue) presented, followed by a probe	Indirect	Reaction to probe indicates attention allocation [144, 145], distinguishing early vs. late attention	Easier setup compared to the visual probe task; distinguishes attention components based on cue-probe timing	Same disadvantages as the visual probe task; relies on inferred attention processes
Visual search task	Participants identify the “odd-one-out” or detect a relevant stimulus among distractors in search matrices [146]	Indirect	(a) Faster detection of relevant stimuli (early attention) and/or (b) increased distraction by relevant stimuli when searching for an irrelevant target (later attention)	Identifies distinct attention components (detection, distraction); can measure attentional approach direction	Does not clarify if distraction is due to inability to shift or attraction to stimuli [50]; high cognitive demand
Eye-tracking	Records eye movements during attention tasks (e.g., food advergame) or free viewing paradigm	Direct	Distinguishes early (fixations/saccades - direction) and late (dwell-time) attention [e.g.^109^]	Captures dynamic attention processes; excellent internal and acceptable test-retest reliability [147–149]; sensitive to temporal components	Expensive equipment; complex data analysis; might not reflect deep cognitive processing
EEG	Measures brain activity and ERPs associated with attention to stimuli during different attention tasks (e.g., attentional blink task, oddball task) or free viewing paradigm	Direct	N170 and P2 ERP components reflect early (automatic) attention [150]; P3/P300 and LPP components reflect late (controlled) attention [151, 152]	Provides precise temporal data on attention; distinguishes automatic vs. controlled processes; reflects motivational attention to cues	Requires specialized equipment and expertise; more intrusive than behavioral methods; more complex interpretation of ERP data

EEG = Electroencephalography; ERPs = event-related potentials; LPP = late positive potential

### Aims

The primary aim of this systematic review is to provide a comprehensive overview of empirical studies which compared ABs to food- and/or body-related stimuli children and adolescents with an ED and those without an ED, and children and adolescents with overweight or obesity and those with healthy weight, using reaction-time methods, ET technology, or recorded ERPs. Furthermore, we would like to review the methodology and quality of existing studies on this topic and compare our results to recent systematic reviews/meta-analyses conducted for related studies in adult samples, if applicable.

## Methods

The systematic review of the literature focusing on ABs to food and body-/shape-related stimuli in children and adolescents with ED symptomatology and healthy comparisons was conducted following the PRISMA guidelines [67] and was registered in the International Prospective Register of Systematic Reviews (PROSPERO; registration ID: CRD42023399292).

### Eligibility criteria

Studies were included when the study: (1) was published in a scientific peer-reviewed journal, (2) investigated ABs for food and/ or body-related stimuli and if AB was explicitly computed, (3) examined a sample of children or adolescents (< 18 years) with either ED symptoms or overweight or obesity and in a healthy (weight) comparison group, and (4) was published in English or German language. Functional magnetic resonance imaging studies were excluded, as they provide high spatial but low temporal resolution and do not allow for direct conclusions about the timing or directionality of attention allocation.

### Search strategy and study selection

Articles were collected by searching the electronic databases PsychInfo, PubMed and Scopus (by MP and TB) on 29th of January 2025. Detailed search terms are available in the additional files.

**Fig. 1:**
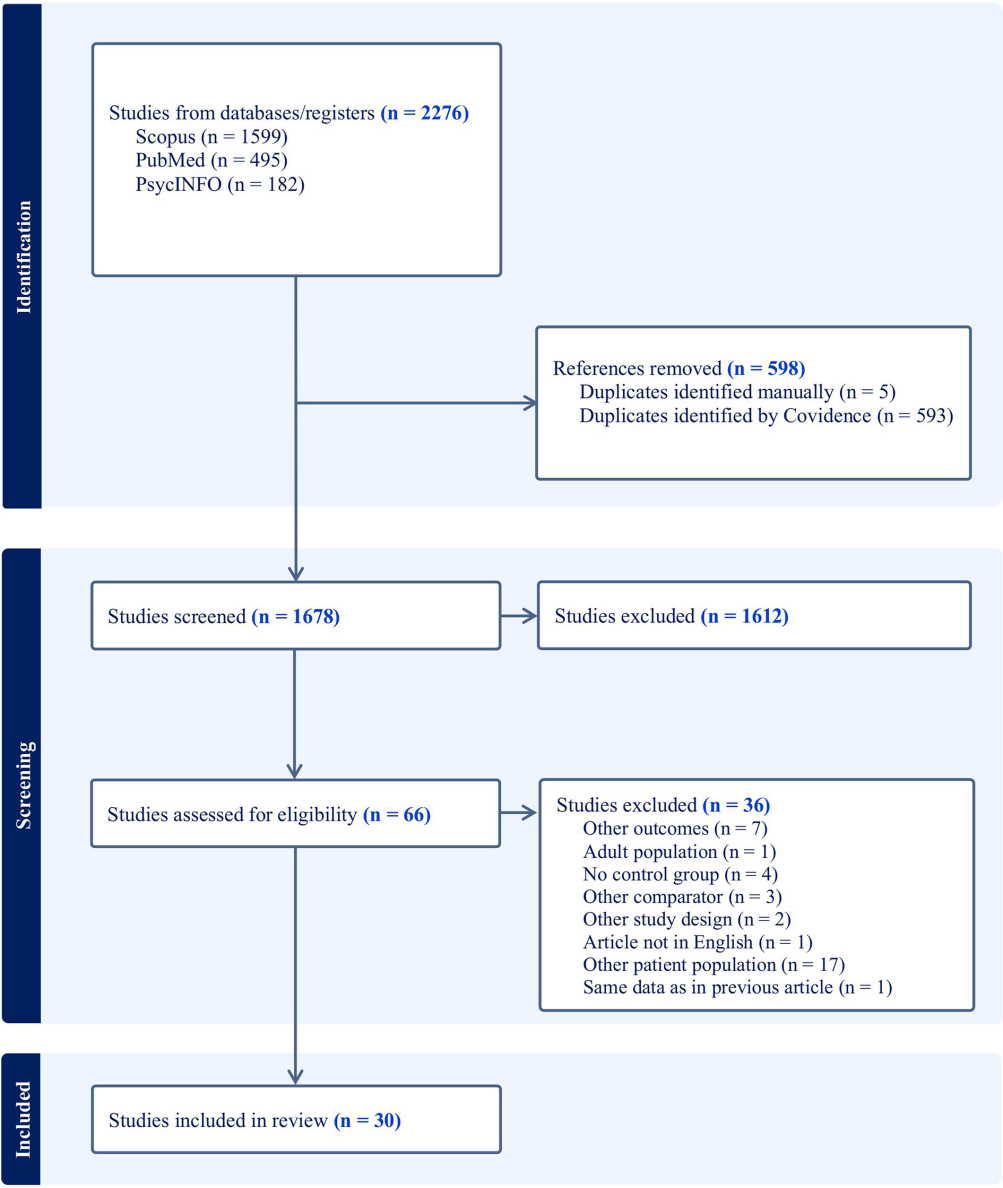
PRISMA flow chart

The results generated from the database search were exported to an online software program to support systematic reviews (Covidence). Duplicates were automatically eliminated by the program. In addition, the list of duplicates was manually reviewed by one reviewer (MP) to ensure that non-duplicates were not erroneously excluded from subsequent stages of systematic review. Independently, two reviewers (MP, JW) then checked the titles and abstracts of the studies and, subsequently, the full text of the studies to establish eligibility. If the reviewers differed in their eligibility rating, they found a consensus after discussion. The studies that were determined to be eligible after this screening procedure were included in the systematic review. For the PRISMA flow chart, see Fig. 1.

The quality of the included studies was assessed using the National Institute of Health quality assessment tool [68], which includes 12 criteria. We focused on criteria concerning study objective, population definition, sample size justification, control selection, case/ comparison group definition, and adjustment for confounding. Criteria related to random selection of participants, use of concurrent controls, timing of exposure assessment, measurement of exposure, and blinding of assessors were not considered, as they were not applicable. Based on the applicable criteria, each study was categorized as good, fair or poor quality by two raters (MP, JW). In case of non-conformity, a consensus was reached after discussion.

## Results

Our literature search yielded a total of 1678 articles and 30 met the criteria for inclusion (see Fig. 1). The results of the studies are summarized in the following section. Table 2 provides an overview of the results of the extracted studies on AB to food-related stimuli; Table 3 contains the results of the studies on AB to body-related stimuli. There were no eligible studies on food-related AB in children or adolescents with BN, nor on body-related AB in those with overweight, obesity, or BED.

**Table 2:** AB to food-related stimuli

Author (s) (year)	Sample^a^	Mean age (SD)	Paradigm	Measure	Attention Bias Index	Stimuli (content)	Findings
Biehl et al. [69]	*N* = 28, Participants with overweight/ obesity (*n* = 14), matched normal weight CG (*n* = 14)	Participants with overweight/ obesity: 12.6 (1.5), matched normal weight CG: 13.5 (1.7)	Free viewing	EEG	ERP (P3)	Pictures^b^ of food (high-calorie, low-calorie) and neutral pictures	Increased P3 for food in all participants; CG: P3 high-calorie foods >P3 low-calorie food OW/OB: no differences depending on calorie value
Hofmann et al. [70]	*N* = 58, Participants with obesity (*n* = 34), HC (*n* = 24)	Participants with obesity: 13.1 (2.16), HC: 14.2 (2.34)	Food picture viewing task	EEG	ERPs (P1 and P3)	Pictures^b^ of food (different degree of processing and calorie content) and non-food stimuli	All participants: P1 and P3 for food >P1 and P3 for non-food
Kösling et al. [71]	*N* = 25, Participants with overweight/ obesity (*n* = 9), and healthy weight (*n* = 16)	Participants with overweight/ obesity: 11.18 (2.16), and healthy weight: 10.63 (1.58)	Free viewing	EEG	Beta band activity	Pictures^b^ of food (high-calorie, low-calorie) and pictures of appealing non-food	Activity in participants with OB/OW >CG (for high-calorie food and appealing non-food)
Woltering et al. [73]	*N* = 40, Participants with obesity (*n* = 22), and normal weight (*n* = 18)	Participants with obesity: 17.7 (1.21), and normal-weight: 15.9 (1.98)	Attention blink task	EEG	Accuracy on attentional blink and ERP (P3)	Pictures of high-calorie food and neutral stimuli	AB task-accuracy in participants with OB < CG; P3: OB < CG
Braet & Crombez [76]	*N* = 74, Participants with obesity (*n* = 34), participants without obesity (*n* = 40)	Participants with obesity: 13.3 (2.0), participants without obesity: 13.9 (2.0)	Stroop task	RT	Differences in response time	Words of food, food-control; emotions, emotions-Control	Response time in participants with OB < CG for food vs. control words; but not for emotion
Fearnbach et al. [72]	*N* = 28, Participants with obesity (*n* = 14), lean participants (*n* = 14)	Participants with obesity: 13.9 (1.1), lean participants: 13.7 (1.1)	Computer-based oddball task	RT + EEG	ERP (P3)	Pictures^b^ of food and non-food Pictures	In participants with OB: neg. correlation btw. body weight, BMI and FM percentage and P3 for food
Rojo-Bofill et al. [77]	*N* = 50, Participants with overweight (*n* = 25), HC (*n* = 25)	Participants with overweight: 10.41 (1.00), HC: 10.32 (1.41)	Dot probe task	RT	Response latency on different stimulus presentation duration (100ms, 500ms and 1500ms)	Pictures^b^ of pleasant food and pleasant non-food	For food vs. non-food; Initial orientation (100 ms): AB in participants with OW = CG; Attentional engagement (500 ms) + maintained attention (1500 ms): AB in participants with OW >CG
Vervoort et al. [78]	*N* = 337, Participants with obesity (*n* = 201), convenience sample (*n* = 136)	Participants with obesity: 14 (2.45), convenience sample: 13 (13.36)	Dot probe task	RT	Interference effects (Incongruent RT – Congruent RT), trial based bias score, probability index	Pictures^b^ of food and neutral stimuli	No effect, participants with OB = CG
Akcay et al. [80]	*N* = 60, Participants with obesity (*n* = 30), and normal weight (*n* = 30)	13.9 (1.26) ^b^	Free viewing	ET	Direction bias and dwell time bias	Pictures^b^ of food (high-calorie, low-calorie) + pictures of other objects	First fixation duration: participants with OB = CG, after 3000 ms maintained A B in participants with OB >CG, especially for high-calorie food
Folkvord et al. [81]	*N* = 95, Participants with overweight (18,6% of the sample), and normal weight (81,4% of the sample)	7.6 (0.54) – 9.6 (0.56)	Food advergame	ET	Gaze duration	Pictures of high-energy snacks or non-food products	AB in participants with OW >CG for food, not for non-food
Werthmann et al. [40]	*N* = 60, Participants with obesity (*n* = 30), and healthy weight (*n* = 30)	Participants with obesity: 11.91 (2.93), and healthy-weight: 11.82 (2.99)	Pictorial visual probe paradigm	ET + RT	Direction bias and gaze duration bias	Pictures^b^ of high-calorie food and of an animal	No effects, AB in participants with OB = CG
Soetens & Braet [79]	*N* = 87, Participants with overweight (*n* = 45), and healthy weight (*n* = 42)	Participants with overweight: 14.98 (1.51), and healthy-weight: 14.74 (1.81)	Imbedded work task	RT	Number of detected words within a time period	High-caloric food- words and control words	No effect, participants with OW = CG
Novosel et al. [83]	*N* = 30, Participants with AN (*n* = 15), HC (*n* = 15)	Age in months: participants with AN: 189.36 (20.07), HC: 188.41 (19.81)	Free viewing	EEG	ERPs (P3 and LPP)	Pictures of pleasant, unpleasent stimuli/ high-calorie, low-calorie food	P300 and LPP for food in participants with AN >CG, AN >CG for low-calorie food
Stonawski et al. [85]	Adults and adolescents (*N* = 39, adolescent participants with AN (*n* = 22), HC (*n* = 17))	Adolescent participants with AN: 15.50 (1.81), HC: 15.75 (1.60)	Free viewing	EEG	ERPs (LPP)	Pictures of neutral stimuli, low-calorie and high-calorie food	All participants: food >neutral stimuli; early LPP findings: Pz: Adolescents with AN >compared to all other groups. P4: Low-calorie stimuli >high-calorie stimuli >neural. Late LPP Findings: Participants with AN (both adolescents and adults) >CG (independent of stimulus type)
Jonker et al. [82]	*N* = 138, Participants with AN and atypical AN (*n* = 69), participants without ED (*n* = 69)	Participants with AN: 15.55 (1.7), participants without ED: 15.48 (1.82)	ARDPEI (Attentional response to distal versus proximal emotional information)	RT	Attentional engagement bias (RT for distal probes in different location as food image – RT for distal probes in same location as food image) – (RT for distal probes in different location as neutral image – RT for distal probes in same location as neutral image) and attentional disengagement bias	Pictures^b^ of high-calorie food and control images	No differences in disengagement, participants with AN = CG, in AN less attentional engagement when brief cues were shown (100ms), no differences in engagement on long time presentation (500ms)
Horndasch et al. [84]	Adults and adolescents (*N* = 36, adolescent participants with AN (*n* = 21), adolescent HC = (*n* = 15)	Adolescent participants with AN: 15.5 (1.9), adolescent HC: 15.5 (1.6))	Free viewing (single stimuli paradigm, one stimulus either low calorie/ high calorie food) and Free Viewing (paired stimuli paradigm both low calorie/ high calorie food matched on visual properties)	ET	Fixation duration (Total fixation time within the ROIs relative to the area of the ROIs of the low calorie and high calorie pictures and total fixation times within the low calorie and high calorie pictures)	Pictures of high-calorie and low-calorie food	In adolescent participants with AN - single stimuli paradigm: AB for high-calorie > low-calorie; paired stimuli paradigm: AB for Low-calorie >high-calorie food
Werthmann et al. [51]	Adults and adolescents (*N* = 65, adolescent participants with AN(*n* = 34), adolescents without AN (*n* = 31))	Adolescent participants with AN: 15.53 (1.46), adolescents without AN: 15.77 (1.26)	Visual dot probe task	RT + ET	Fixation direction bias and fixation duration bias	Pictures^b^ of high-calorie, low-calorie food and non-food items	Adolescents with AN = CG direction + maintenance of attention to food (especially high calorie)
Schmidt et al. [86]	*N* = 50, Participants with BED (*n* = 25), healthy participants without ED (*n* = 25)	Participants with BED: 14.68 (2.85), healthy participants without ED: 15.28 (2.39)	Task 1: Free viewing (free exploration) Task 2: Visual search task	ET + RT	Gaze duration bias, gaze direction bias and detection time (mean RT of trials including a non-food target among food distractors - the mean RT on trials including a food target among non-food distractors from)	Pictures of food (high, low and medium calories) and non-food items	Task 1: inparticipants with BED >CG for gaze duration, in participants with BED = CG for gaze direction; Task 2: in participants with BED >CG (faster detection)
Shank et al. [89]	*N* = 89, Participants with LOC (*n* = 47), participants without LOC (*n* = 42)	Participants with LOC: 13.8 (2.4), participants without LOC: 15.6 (1.6) (sign. difference in age)	Dot probe Task	RT	Three RT biases: RT to the less salient image in the pair - RT to the more salient of the paired images (for high-palatable vs. low-palatable food vs. non-food)	Pictures^b^ of palatable, less palatable food and non-food items	Participants with LOC = CG, but in participants with LOC higher BMI →AB to high palatable foods vs. non-food
Van Malderen et al. [90]	*N* = 295, participants with LOC (*n* = 93), participants without LOC (*n* = 202)	Participants with LOC: 13.79 (2.05), participants without LOC: 13.23 (1.94)	Dot probe Task	RT	RT bias: RT non-food – RT food	Pictures^b^ of food and neutral non-food pictures	Overall significant AB toward food; interaction between weaker regulatory control and AB increased odds of reporting LOC
Van Malderen et al. [91]	*N* = 80, Participants with LOC (*n* = 23), participants without LOC (*n* = 57)	Participants with LOC: 13.74 (1.96), participants without LOC: 13.09 (1.91)	Dot probe task	RT	RT bias: RT non-food – RT food	Pictures^b^ of food and neutral non-food pictures	Significant three-way interaction between inhibitory control, AB, and LOC; in participants without LOC, poor inhibitory control and low AB linked to unhealthy food choices; no significant interaction between inhibitory control and AB in predicting unhealthy food choices for adolescents with LOC

AB = Attentional Bias, AN = Anorexia Nervosa, BED = Binge-Eating Disorder, CG = Comparison Group, ED = Eating Disorder, EEG = Electroencephalography, ET = Eye-Tracking, HC = Healthy Control, LOC = Loss of Control Eating, LPP = Late Positive Potential, OB = Obese, OW = Overweight, ROIs = Regions Of Interest, RT = Reaction Time, ^a^= Sample descriptions are reported as in the original studies, ^b^= Pictures from databases like Blechert et al. [153], International Affective Picture System (IAPS) or previously piloted photo dataset

**Table 3:** AB to body-related stimuli

Author (s) (year)	Sample^a^	Mean age (SD)	Paradigm	Measure	Attention bias index	Stimuli (content)	Findings
Bauer et al. [94]	*N* = 99, Participants with AN (*n* = 56), HC (*n* = 43)	Participants with AN: 16.09 (1.03), HC: 15.85 (1.77)	Free viewing	ET	Relative fixation time compared between time intervals (1000-6000ms) of the overall presentation time	Pictures of own body	Early: participants with AN to “unattractive” areas > CG; over time decreased, but remained higher than CG
Pinhas et al. [95]	*N* = 33, Participants with AN (*n* = 13), HC (*n* = 20)	Participants with AN: 14.5 (1.61), HC: 14.4 (1.82)	Free viewing	ET	Relative fixation duration	Pictures of thin body stimuli, positive social interaction, overweight body stimuli and neutral objects	In participants with AN: longest fixations for thin > overweight > social interaction
Sfärlea et al. [96]	*N* = 72, Participants with AN (*n* = 28), participants with MD (*n* = 20), HC (no mental illness, *n* = 24)	Participants with AN: 15.37 (1.36), participants with MD: 16.37 (1.14), HC: 16.43 (1.56)	Free viewing	ET	Percentage of first fixation and Percentage of dwell time	Pictures of normal weight bodies, neutral expression faces^b^, a underweight body, an overweight body, a happy face and an angry face	For initial orientation: no differences, for maintenance: all groups AB body > face; in participants with AN: more pronounced (esp. for underweight)
Svaldi et al. [97]	*N* = 24, Participants with AN (*n* = 12), CG without lifetime ED (*n* = 12)	Participants with AN: 15.14 (1.55), HC: 15.14 (1.57)	Free viewing & Mood Manipulation (negative vs. positive mood induction, within cross-over design)	ET	Gaze duration	Viewing pattern of own body in mirror	In participants with AN: AB for “ugly” > ”beautiful”, In CG: only in neg. mood induction
Horndasch et al. [98]	*N* = 31, Participants with AN (*n* = 13), Typically developing adolescent girls (*n* = 18)	Participants with AN: 15.7 (1.8), typically developing adolescent girls: 16.6 (1.8)	Free viewing	EEG	ERPs (earlier and later LPP)	Pictures of 3 Body Schemes	In participants with AN: highest LPP for underweight, in CG: highest LPP for overweight, over time shift to normal weight
Horndasch et al. [99]	Adults and adolescents (*N* = 35, adolescent participants with AN (*n* = 19), comparison participants (*n* = 16))	Adolescent participants with AN: 15.2 (1.8), comparison participants: 16.3 (1.2)	Free viewing	EEG	ERPs (LPP)	Pictures of women’s bodies in underwear from different BMI categories	For all participants: earlier posterior components: highest amplitudes for extremely overweight stimuli; central LLP: focus on underweight stimuli
Romero Frausto et al. [100]	Adults and adolescents (*N* = 68, adolescent participants with AN (*n* = 26), HC (*n* = 42))	Adolescent participants with AN:15.33 (1.65), HC = 15.98 (1.81)	Free viewing	EEG	Cluster analysis (50-550ms)	Pictures of computer-generated women bodies in underwear from different BMI categories	In adolescents with AN: stronger linear and quadratic trends and (numerically) relatively higher neural activity in response to underweight and relatively lower neural activity in response to higher-weight body pictures
Bauer et al. [92]	*N* = 141, Participants with different types of ED (AN-R *n* = 30, AN-BP *n* = 26, BN *n* = 22), HC (*n* = 43), clinical controls with anxiety disorders (*n* = 20)	Participants with AN-R: 15.80 (1.09), with AN-BP: 16.42 (0.85), and with BN: 16.72 (0.76), clinical controls: 15.94 (1.64), HC: 15.85 (1.77)	Free viewing	ET	Fixation duration	Pictures of own vs. female peer body	All subgroups of ED: AB for unattractive areas on own body > peer body; in participants with AN-R: AB for unattractive than on attractive compared to controls
Horndasch et al. [93]	*N* = 42, Participants with an ED (*n* = 17 overall: AN (*n* = 11), BN = 6), adolescents without current or history of psychiatric disorder, high degree of ED symptoms or psychopharmacological medication (*n* = 25)	Participants with ED: 16.00 (1.6) (AN: 15.4 (1.8), BN: 17.2 (1.6)) / Control: 15.3 (1.9)	Free viewing	ET	Total fixation time within each ROI was calculated and corrected for the size of the bodies (relative to the whole picture) and the specific body parts (relative to all ROI).	Pictures of 3 body schemes (underweight, normal-weight, overweight)	For “Index-area”: participants with ED = CG, in normal weight: AB in participants with ED > CG for unclothed body parts

AB = Attentional Bias, AN = Anorexia Nervosa, AN-BP = Anorexia Nervosa Binge-Eating/ Purge, AN-R = Anorexia Nervosa Restricting, BN = Bulimia Nervosa, CG = Comparison Group, ED = Eating Disorder, EEG = Electroencephalography, ERP = Event Related Potential, ET = Eye-Tracking, HC = Healthy Controls, LPP = Late Positive Potential, ROIs = Regions Of Interest, ^a^= Sample descriptions are reported as in the original studies, ^b^ = Pictures from databases like Karolinska Directed Emotional Faces

In this manuscript, all individuals who were compared with the respective target population (ED symptoms or obesity/overweight) are continuously termed as “comparison group”. Across the studies reviewed, the definition and composition of comparison groups varied widely. For clarity, we have applied the term “comparison group” as a general umbrella term, which may differ from the specific terminology used in individual studies; exact specifications for each study are provided in Tables 2 and 3 along with further details on the results. Furthermore, when referring to children and adolescents collectively, we will use the term “young people” throughout the manuscript.

### Ratings of study quality

The study quality was rated as poor in three studies, as fair in seventeen studies, and as good in ten studies. The weakest ratings were achieved in the category sample size justification and report of confounding variables. In contrast, most studies achieved strong ratings in the category clarity and relevance of research objectives. A detailed overview of the overall ratings can be found in the additional files.

### AB to food-related stimuli in young people with overweight/ obesity

Twelve studies examined AB toward food-related stimuli in young people with overweight or obesity. Among these, five studies analyzed EEG ERPs, three studies investigated RTs, one used an IWT and three studies employed ET to assess eye movements as indicators of AB.

#### Electroencephalography event-related potentials studies

Three of the identified studies used a free viewing task during EEG [69–71], one study analyzed EEG data and RTs [72] and one used an attention blink task while EEG was recorded [73]. In the study by Woltering et al. [73] the response data of the adolescent group with obesity showed lower accuracy on attentional blink task to food stimuli (high-calorie food (e.g., burger) vs. neutral stimulus (e.g., chair)) compared to the comparison group, suggesting their attentional resources were more engaged by food. Additionally, the P3 component was smaller during the attentional blink when food (vs. neutral) pictures were presented, indicating a reduced ability to update working memory once attention was captured by food stimuli in adolescents with obesity. Kösling et al. [71] found that children with overweight or obesity, compared to the comparison group, showed significantly increased relative activity in the central beta band in response to high-calorie foods and appealing non-food stimuli (such as kittens or emojis), but not low-calorie foods. Elevated beta band activity is typically associated with increased perceptual effort and heightened focus on stimuli [74, 75]. One interpretation of this finding is that in children with obesity more perceptual resources were occupied by appealing as well as high calorie food cues indicating that children with obesity may exhibit greater cognitive engagement and attentional focus towards these stimuli than the comparison group. Together, these studies suggest that young people with overweight or obesity experience a higher cognitive load when confronted to rewards than comparisons, as evidenced by increased engagement and attentional focus on food and appealing stimuli in young people with overweight or obesity. Supporting this finding, Fearnbach et al. [72], found negative correlations between indicators of obesity (body weight, BMI, and fat mass percentage) and the P3 amplitude difference between food and neutral stimuli (household) after a 45 min cycling exercise in a computer-based oddball task. This finding may suggest that motivation towards food diminishes after having exercised in adolescents with obesity, but not in comparisons. In contrast, the studies by Biehl et al. [69] and Hofmann et al. [70] highlight the role of impulsivity and motivation in shaping the attentional bias for food. Biehl et al. [69] found increased P300 amplitudes for food vs. neutral (office supplies) images in adolescents, irrespective of their body weight, suggesting generally an increased allocation of attention towards food cues in adolescents. The comparison group showed significantly higher P300 amplitudes when viewing high-calorie foods vs. low-calorie foods or neutral items. In contrast, adolescents with overweight/ obesity showed no difference in P300 amplitudes based on the caloric content, although they still distinguished between food and non-food images. Hofmann et al. [70] reported comparable results using food stimuli that varied in the degree of food processing and calorie content (e.g., chocolate, crisps, broccoli, tomato) compared to non-food stimuli (e.g., cup, screwdriver). All participants, irrespective of their weight status, preferentially attended to processed food cues vs. non-food cues in the early (P100) and late (P300) ERP components. Correlational analyses, however, yielded, that impulsivity correlated positively with food-specific P100 amplitudes, across and within, both groups.

#### Reaction time studies and imbedded word task

Three studies assessed RT as a measure of AB in different tasks and stimulus cues [76–78]. Soetens & Braet [79], who used an IWT, and Vervoort et al. [78] found no evidence of AB towards food stimuli. In the study by Soetens & Braet [79], no attention interference effects for high-calorie food words (vs. control words) were found, neither for adolescents with overweight nor for the comparison group. Similarly, Vervoort et al. [78], using RT assessments for three attention indices, reported no significant differences in AB for food vs. neutral stimuli between adolescents with obesity and the comparison group. In contrast, the study by Rojo-Bofill et al. [77] identified a differential attention pattern for food vs. non-food cues in children with overweight compared to controls, depending on the duration of stimulus presentation. During initial orienting (100-ms presentation rate), both groups exhibited a bias towards food vs. non-food images compared. However, at longer presentation rates (500-ms and 1500-ms), only the children with overweight demonstrated biased attention engagement and maintained attention towards food cues. This indicates that while biased initial orienting to food cues occurred regardless of weight, sustained ABs were specific to children with overweight. Braet and Crombez [76] also reported differences in attention for food vs. non-food words in children with and without obesity. Using a Stroop task with food, non-food/control, emotion, and non-emotional/control word stimuli, they found that children with obesity were slower at color-naming food words compared to non-food/control words – a pattern not observed for emotion words or in the comparison group. One interpretation of this finding is that in children with obesity, AB is specifically linked to food-related cues rather than to negative emotional stimuli.

#### Eye-tracking studies

Akcay et al. [81], Folkvord et al. [82] and Werthmann et al. [41] used ET to investigate eye movements as an indicator of food-related AB in young people. Akcay et al. [80] measured AB towards low-calorie foods (e.g., salads, fruits and vegetables), high-calorie foods (e.g., high-fat meat, sweets and salty snacks) and non-food neutral objects. Adolescents with obesity looked significantly more often towards food (i.e., direction bias) and maintained their attention longer on food images, especially high-calorie foods, than on non-food images compared to the comparison group. This difference was most pronounced after 3000 ms stimulus presentation duration, suggesting adolescents with obesity process food-related stimuli differently, showing a greater tendency to direct and maintain attention on food cues. Likewise, Folkvord et al. [81], in which children played an advertising game promoting either high-energy snacks or non-food products, found that children with overweight maintained their attention longer to the food cues than the comparison group, with no group differences for non-food products. In contrast, Werthmann et al. [40] did not find significant group differences in food-related AB. All children, regardless of weight status, had a tendency to orientate toward palatable high-calorie food cues (i.e., direction bias) and initially maintain their first gaze longer on food cues than on nonfood cues (i.e., initial gaze duration bias).

### AB to food-related stimuli in young people with EDs

Five studies assessed AB to food in young people with AN, one study investigated ABs to food stimuli in adolescents with BED and three studies examined the AB to food in adolescents with loss of control (LOC) eating compared to a comparison group.

#### AB for food in young people with AN

Two studies which investigated AB in young people with AN measured ERPs [84, 85], one study relied on RT, using an ARDPEI [82], and two studies relied on ET [51, 84]. The study by Novosel et al. [83]. , which analyzed ERPs during a free-viewing paradigm, found that adolescents with AN showed stronger neural response to food stimuli—particularly low-calorie foods—than the comparison group. This was evident in larger P300 amplitudes (240–380 ms) reflecting enhanced early attention allocation and greater LPP amplitudes (400–700 ms) indicating sustained attentional processing. No significant differences were observed for high-calorie foods [83]. Similarly, Stonawski et al. [85] examined the neural processing of food stimuli in adolescents and adults with AN using ERP measurement. They found that both adolescents and adults (participants with AN and comparison group) showed larger early LPP amplitudes (350-700ms) in response to food compared to neutral stimuli, with adolescents showing higher early LPP amplitudes than adults at P3 and the highest amplitudes at Pz in adolescents with AN. In the late LPP phase (800-1200ms), participants with AN showed larger amplitudes than the comparison group, regardless of age, across all electrodes and for all stimulus categories, indicating more intensive cognitive stimulus processing. The study by Jonker et al. [82] used the ARDPEI-task, which was specifically designed to distinguish between attentional engagement with and disengagement from food stimuli. They found no differences in attentional disengagement for high-calorie food pictures versus control pictures (i.e., office items, household items, art items) between adolescents with AN and the comparison group. Adolescents with AN showed reduced engagement towards food pictures, particularly at short stimulus presentation times [82]. Horndasch et al. [84] further investigated w AB in AN depends on the type of food presented (i.e., presentation content) and whether competing stimuli are available to attract attention (i.e., stimulus competition). Their results demonstrated increased fixation times for high-calorie food relative to the fixation times for low-calorie food in participants with AN compared to comparison participants only in trials when high-calorie food or low-calorie food were presented alone, without a competing stimulus. However, when high-calorie foods and low-calorie foods were presented simultaneously, a visual attention shift towards low-calorie and away from high-calorie stimuli was observed in participants with AN. Note that the sample consisted of adolescents and adults and results were not reported separately per age group. Complementing these results, Werthmann et al. [51] found that both adolescents with AN and the comparison group exhibited significantly increased direction and duration bias to food cues, particularly for high-calorie foods. However, a significant bias toward low-calorie food was observed exclusively in adolescents with AN and this low-calorie food bias was not observed in adults with and without AN and adolescents without ED symptoms. This observation is in line with the findings of Horndasch et al. [84] regarding the attention preference of participants with AN for low-calorie food when presented with competing stimuli and also aligns with observation of Novosel et al. [83]. of the greater AB toward low-calorie food in participants with AN compared to the comparison group.

#### AB for food in young people with BED and loss of control eating

We identified only one study in our review testing food-related AB in young people with BED. Adolescents with BED had a significantly longer attention duration on food vs. control images (i.e., high, low, and medium calorie foods vs. an office or household tool, or nature object) compared to a comparison group based on ET data in a free exploration paradigm [86]. Interestingly, both groups did not differ in gaze direction bias. In the same study, another attention paradigm was employed, namely a visual search task with RT assessment, which allowed for identifying processing of multiple competing, yet perceptually similar, stimuli. Here, adolescents with BED showed a detection bias in recognizing food vs. non-food stimuli relative to comparison participants, which suggests a faster detection of food cues among an array of other cues. In summary, in the only study testing young people with BED, a duration bias and a detection bias for food vs. non-food cues was found in adolescents with BED relative to the comparison group, indicating a pronounced motivational relevance of food for young people with BED.

Three studies investigated ABs to food stimuli in young people with loss of control (LOC) eating relative to a comparison group. LOC eating can be a preliminary state of clinical EDs such as BN or BED [87], and prevalence rates suggest that LOC is a common experience among adolescents [88] and therefore we included these studies in the present review.

All three studies used a dot-probe task with food and control stimuli [89–91]. Shank et al. [89] did not find significant differences in AB for palatable and less palatable foods in adolescents with LOC vs. the comparison group. Interestingly, a significant interaction of BMI-z score and LOC eating for AB towards palatable foods was reported: in the comparison group, higher BMI was associated with attentional avoidance of palatable foods. In contrast, for participants with LOC, a higher BMI was associated with an AB towards palatable foods.

Van Malderen et al. [90, 91] conducted two studies about LOC in adolescents in community samples. In the first study, all participants had an AB towards food images, irrespective of LOC status. However, results yielded an interaction indicating that a combination of increased behavioral reactive processing (AB) and weaker inhibitory control significantly increased the odds of reporting LOC. Extending these results, the second study by [91] tested the interaction between inhibitory control and reactive processes (i.e., AB) to predict (un)healthy food choice in adolescents. A significant three-way interaction between inhibitory control, AB and LOC emerged: In adolescents without LOC, a combination of poor inhibitory control and low AB was significantly associated with unhealthy food choices. Unexpectedly, there was no significant association between unhealthy food choices and inhibitory control or AB in adolescents with LOC.

Thus, overall, there is initial evidence for a preferential attention processing of food cues in young people with BED, yet evidence on the association of the (more subclinical) LOC and food-related attention remain inconsistent.

### AB to body-related stimuli in young people with EDs

Seven of the included studies examined AB to body-related stimuli in young people with AN. Two studies examined AB to body-related stimuli in young people with different ED diagnoses [92, 93].

#### AB for body-related stimuli in young people with AN

Four of the studies which examined AB to body-related stimuli in young people with AN used ET [94–97], three studies used ERPs [98–100]. Two ET studies examined gaze behavior towards participants’ own bodies [94, 97], the other examined gaze behavior regarding bodies with different body proportions (different BMI categories or underweight, normal weight or overweight bodies [95, 96, 98–100]). Two of these studies [95, 96] social interactions and facial expressions as comparison stimuli.

In the study by Bauer et al. [94], adolescents with AN showed initially a significantly stronger tendency to focus on body areas that were subjectively perceived as unattractive. Over the whole picture presentation time (6s), however, their attention to these body parts decreased. Nevertheless, the AB scores remained consistently higher in adolescents with AN than in the comparison group throughout the stimulus presentation. This corroborates with results by Svaldi et al. [97], examining gaze duration on participants’ own body parts after positive or negative mood induction. The expected 3-way interaction (group: AN/HC; mood: negative/positive; body part: beautiful/ugly) did not reach significance. Yet, post-hoc tests demonstrated that adolescents with AN consistently focused longer on their perceived ugliest body part compared to the most beautiful one, regardless of mood induction conditions (positive or negative). Participants in the comparison group also focused longer on their ugliest body part, but only in the positive mood condition. The authors explain that the tendency of adolescents to focus longer on body parts they perceive as unattractive may reflect the overvaluation of shape and weight typical of this developmental period, in which the body undergoes significant physical changes. The study of Pinhas et al. [95] provided additional insights by comparing gaze behavior toward body shapes and social interactions. When “thin”, “overweight”, and “social interaction” images were shown together, participants with AN exhibited the longest fixation on “thin” body shapes, followed by “overweight” images, with social interactions stimuli receiving the least attention. This pattern was not observed in the comparison group. This indicates a strong bias in participants with AN towards body-related stimuli, particularly those that align with their ideal thin body image. This is in line with findings by Sfärlea et al. [96]: In this study ABs were assessed in a free-viewing task in which female bodies and faces were presented simultaneously and thus competed directly for attentional resources (neutral trials: neutral faces and normal weight bodies, emotional trials: underweight/overweight body and happy/sad faces). All groups (adolescents with AN, adolescents with major depression and the comparison group) showed a duration bias for bodies (in both the neutral and the emotional trials) compared to face stimuli. Yet, this bias was more pronounced in adolescents with AN than in participants with depression and the comparison group, especially when viewing underweight bodies.

Supporting these findings, three EEG studies have shown that participants with AN exhibit the highest LPP amplitudes when viewing underweight body images compared to normal-weight and overweight bodies, in both early and later time windows after stimulus onset: Romero Frausto et al. [100] examined visual body size estimation in adolescents with AN. Participants first completed a viewing task, followed by two depictive body size estimation tasks, during which body images across six BMI categories (underweight to overweight) were presented for 800 ms. EEG-analysis results that adolescents with AN exhibited relatively higher neural activity in response to underweight bodies and lower activity to higher-weight bodies compared to the comparison group. This is consistent with Horndasch et al. [98]: in an earlier as well as in a later time window LPP amplitudes of participants with AN were highest when looking at pictures of underweight compared to women with normal-weight and overweight, suggesting that underweight stimuli capture and maintain attention in participants with AN significantly more than normal-weight or overweight stimuli. In contrast, the comparison group initially showed the highest amplitudes in response to overweight images, with a shift towards normal-weight images over time, indicating a more balanced processing of body weight. Another study by Horndasch et al. [99] found similar results: the comparison group, but also adolescents with AN, exhibited the highest amplitudes for extremely overweight stimuli regarding earlier posterior ERP components (450-700ms). However, the central LPP (1000–1300 ms) revealed a general focus on underweight across all adolescents. As adult participants were included in the study, the cross-sectional data indicated that with increasing age and illness duration, participants with AN exhibited more pronounced alterations in gaze behavior, with an intensified focus on underweight bodies compared to the comparison group.

#### AB for body-related stimuli in adolescents with different types of EDs

The studies by Bauer et al. [92], including participants with AN-restrictive type (AN-R) AN-binge eating/purging type (AN-BP), and BN and Horndasch et al. [93], who examined AB in participants with AN and BN, both highlight a pattern of AB towards body areas perceived as unattractive. In the study of Bauer et al. [92], participants looked at pictures of themselves and a female peer’s body. All participants, regardless of diagnosis, preferentially attended to body areas perceived as unattractive, especially self-defined unattractive areas of their own body. This pattern was most pronounced in girls with ED, particularly those with AN-R, who fixated significantly longer on unattractive and shorter on attractive areas than controls. The AN-BP and BN subgroups did not differ significantly from controls.

Horndasch et al. [93], who examined AB towards three body types (underweight, normal weight, overweight) in a swimsuit or underwear as stimulus material, found a main effect of “index areas” (weight-indicating areas such as hips, abdomen). Both, participants with ED and comparison group, focused longer on index areas than on the rest of the body, with the longest fixation times on the index areas for the stimulus category “normal weight”. However, participants with an ED fixated significantly longer on unclothed than on clothed body parts compared to controls.

## Discussion

The purpose of the present study was to conduct a systematic review of ABs to food- and body-related stimuli in young people with EDs compared to those without EDs, and in those with overweight or obesity compared to those with healthy weight.

ABs may contribute to their development or indicate risk by reinforcing maladaptive behaviors and perceptions. As attentional processes may be influenced by developmental factors that may differ from those in adults, investigating these biases in younger populations may offer insights relevant for early prevention and intervention.

### AB to food-related stimuli in young people with overweight/ obesity

Our review of the research on AB for food in young people with overweight and obesity reveals a heterogeneous picture. One study found children with obesity have a stronger AB towards food [81], while another indicated differences in attention with regard to food only in late information processing in children with overweight [77]. Additionally, a recent study revealed that children with obesity pay increased attention to all reward stimuli, not just food [71]. In adolescents some studies reported an AB for food in adolescents with obesity compared to the comparison group, while others found no significant differences in AB for food vs. neutral stimuli between adolescents with obesity and the comparison group. This inconsistency mirrors findings of reviews in adults, where early research indicated a clear AB toward high-calorie foods, but as more studies were conducted, the picture became less consistent [20, 43, 101]. Taken together, the findings on food-related AB in young people with overweight and obesity are contradictory and the evidence for an AB for food is poor.

### AB to food-related stimuli in young people with AN

Our review of research findings on ABs in adolescents with AN revealed complex and nuanced patterns in their responses to food-related stimuli. When an AB was observed, adolescents with AN tended to focus more on low-calorie foods. According to the theory of “motivated attention”, the increased attentional response to low-calorie food may indicated motivational importance of these foods for adolescents with AN [102]. Other findings paint a more differentiated picture. Early attentional engagement with food cues seemed reduced in adolescents with AN compared to controls, particularly when stimuli were presented very briefly, while attentional disengagement did not differ consistently. Attentional patterns for high-calorie foods were inconsistent and seemed to depend on factors such as whether high- and low-calorie foods were presented simultaneously or in isolation. These findings partially relate to the conclusions of Stott et al. [103], whose meta-review of adult ED populations reported variable patterns of attentional vigilance and avoidance depending on stimulus properties. However, in contrast to the clearer patterns observed in adult samples, the findings on AB to food-related stimuli in young people with AN remain inconsistent, and the overall evidence base for a consistent AB in this population is weak.

### AB to food-related stimuli in young people with BED/LOC-eating

The review of studies on AB in young people with BED and LOC suggested a heightened attentional sensitivity to food-related stimuli in BED, but a more heterogeneous picture regarding LOC. The only study in adolescents with BED [86] found pronounced AB for food (i.e., duration and detection bias), indicating that AB for food may be relevant in individuals with BED. As both the BED and the comparison group had similarly elevated BMI, these findings suggest that the observed AB cannot be explained by overweight alone. While consistent with evidence from adults showing heightened AB for food in BED [104], these results should be treated with caution and regarded as very preliminary, as they are based on a single study.

Findings in LOC eating were more heterogeneous. Shank et al. [89] reported no overall group differences in AB, but noted an interaction between BMI and AB: in controls, higher BMI was linked to attentional avoidance of palatable foods, whereas in participants with LOC, higher BMI was associated with an increased AB towards these foods. This suggests that BMI and AB interact differently depending on LOC, indicating a non-linear relationship influenced by eating control mechanisms [105]. Future research should therefore investigate the potential overlap between LOC eating and attentional processes in individuals with higher BMI, like in overweight and obesity. The studies by Van Malderen et al. [90, 91] highlighted an interaction between such weak regulatory processes and stronger reactive responses (AB), increasing the odds of LOC eating. However, AB and inhibitory control did not significantly predict unhealthy food choices in adolescents with LOC, unlike in comparisons where poor regulation and low AB were linked to unhealthy choices. According to the authors, the current results indicate that vulnerability has no additional explanatory value in relation to food choices. Again, the results on AB to food stimuli in young people with LOC eating are inconclusive, and the evidence for an AB to food in young people with LOC eating is weak.

### AB to body-related stimuli in young people with AN

Across all studies included in this review, an AB for different body-related stimuli in adolescents with AN was found, regardless of the measurement method. ET research revealed that adolescents with AN tend to focus initially on body parts they perceive as unattractive, a pattern that is not seen in the comparison groups [94, 97, 106]. These results are in line with previous studies in adults demonstrating that women with EDs tend to pay more attention to self-defined unattractive body areas [107–109]. In adolescents, with AN there is also a sustained focus on “thin” body shapes over “fat” body shapes and social interactions, with these results suggesting “dual” attentional biases in adolescents with AN (i.e., towards thin bodies and away from emotional faces), reflecting the pronounced motivational preference for thin-ideal in adolescents with AN [95].

The three EEG studies we reviewed further complement these findings by showing that individuals with AN exhibit increased LPP amplitudes in response to images of underweight individuals [98–100]. In contrast, the comparison groups display increased neural responses to images of overweight individuals. In summary, the results indicate that adolescents with AN show increased attention to underweight body types, whereas the comparison groups show increased attention to overweight bodies. To summarize, the existing research indicates that young people with AN have an AB towards thin bodies, as evidenced by several studies using different types of attention indices. However, it also should be noted, that the evidence for AB to body-related stimuli young people with AN must be regarded as provisional due to the small number of studies, very small samples and diverse measurement instruments as well as the diverse stimuli sets employed.

### AB to body-related stimuli in young people with different types of EDs

The two included studies about AB to body-related stimuli in young people with different types of EDs reveal focused attention on body areas perceive as unattractive, as well as for the participants with EDs and the comparison groups. Bauer et al. [92] found that all participants, regardless of the diagnosis of an ED, focused more on body areas they perceive as unattractive, especially on their own bodies. This bias was stronger in girls with ED, particularly those with AN-R, who spent more time looking at unattractive areas and less at attractive areas than controls, consistent with similar findings in studies that included only participants with AN.

Horndasch et al. [93] also reported the tendency to fixate on ‘index areas’ (e.g., hips, abdomen—regions often associated with body dissatisfaction or weight perception) for participants with ED and comparison groups. While both groups focused on these areas, especially for normal weight stimuli, participants with ED showed increased AB to unclothed body parts. In summary, the comparison of a group of young people with different EDs (including participants with AN) and comparison groups shows no specific evidence for AB to body-related stimuli, although the level of evidence is considered weak due to the small number of studies and sometimes very small samples.

### Methodological considerations and future directions

The findings of this review highlight considerable heterogeneity across studies, particularly regarding AB for food stimuli. These inconsistencies might be attributable to several factors, including methodological limitations. The overall number of studies is small and many of the reviewed studies are based on small sample sizes, which limits the generalizability of the results. In addition, the methods used to assess ABs—whether direct (e.g., ET) or indirect (e.g., Stroop or dot-probe tasks)—lead to a certain degree of variability of results. For instance, ET results depend on the reliability of area-of-interest (AOI) markings, which may lack precision for neighboring or very small AOIs and often rely on manufacturer-provided metrics rather than empirically validated measures [110]. Indirect methods also suffer from low test-retest reliability and internal consistency, further complicating comparisons across studies [111]. When these psychometric properties are inadequate, the validity of group comparisons may be compromised. This can occur, for example, due to insufficient specificity or sensitivity, when “true” group differences are obscured by an unreliable measurement, or when the measurement captures variance attributable to other factors rather than to the construct of interest. As reporting on the reliability and internal consistency of AB measures is scarce [112, 113] these limitations warrant careful consideration when interpreting existing findings.

Differences in stimulus selection, like comparing high- and low-calorie foods or underweight and overweight bodies, could also affect the context in which stimuli are evaluated, as noted by Werthmann et al. [20]. Additionally, factors like image brightness, contrast, or palatability can influence AB [114], although not all studies included in this review took this into account. Piloting stimuli sets taking these influences into account would be essential and helpful.

Additionally, most research has focused on specific EDs, like AN, or groups with overweight/obesity, with limited studies examining the full diagnostic spectrum. For example, very few studies have included participants with BED and BN. We included studies on LOC eating because it shows considerable symptom overlap with BED in the current diagnostic framework [115] and is often studied in children and adolescents, where determining objective binge size is difficult [116–118]. However, we acknowledge that the inclusion of LOC may reduce diagnostic specificity, and this limitation should be considered when interpreting the results.

Given these possible explanations for the inconsistent findings, some potential directions for future research and implications for treatment are discussed below. First, addressing methodological limitations is crucial for future studies. For example, future research could use images from standardized data sets, such as food-pics [119], to ensure consistency and reliability in stimuli. Additionally, based on recent findings regarding anxiety and substance-related ABs in adults, incorporating a novel dual-probe variant of the attention probe task could be beneficial, in which two probes are briefly presented on each trial, and attentional distribution is inferred from relative accuracy to identify probes appearing in each screen location [120]. This novel dot probe approach, which offers high psychometric reliability, could enhance realism by using video stimuli instead of static images [120–122].

Extending research to other population groups is another important research approach. There is a notable lack of studies looking at body-related attention in children or adolescents with a higher body mass index (BMI), overweight or obesity, even though research with adults suggests such an AB [123], which has been associated with the development and maintenance of body dissatisfaction and eating disorders [59]. Dissatisfaction with one’s own body as a psychological distress is also associated with obesity in young people [124], highlighting the need for further research in this population to support early prevention measures. In addition, including a wider range of EDs in future studies will provide a more comprehensive understanding of body-related biases. Currently, there is a lack of research investigating AB towards body-related stimuli in other EDs, such as BN.

Furthermore, many studies do not take various confounding variables into account, such as duration of illness, gender, age, hunger and medication, as psychotropic medication may influence eye-movements (e.g, [125]).

A stronger experimental psychopathology approach is essential to advance our understanding of the role of AB in EDs. Specifically, experimental research should examine whether targeted interventions to manipulate AB can reduce symptoms in the short term. Although some studies have already addressed this question, they could not be included in the present review because they lacked a comparison group without EDs, highlighting the need for future research [126–128]. Additionally, longitudinal research is needed to assess whether AB shifts during treatment and how these changes relate to symptom improvement [129].

Such studies would provide direct evidence for the causal role of AB in the maintenance or exacerbation of EDs and would bridge the gap between correlational findings and clinical applications, offering a more robust foundation for developing AB-focused therapeutic strategies. For example, in AN, therapeutic strategies could focus on promoting a more balanced and flexible attentional response to different types of food stimuli. Interventions such as food exposure could support a more neutral and less selective processing of food cues, thereby fostering a more general approach and acceptance of food [130, 131]. With regard to body-related AB, exposure therapy with the patient’s own body, such as mirror exposure therapy, has been used effectively in adults [132, 133] and could therefore also be used more frequently in the treatment of young people with ED. In this context, a change in AB is being discussed as a mechanism of action [133–135]. Since body dissatisfaction is frequently associated with overweight and obesity, and studies in adults often find a link between this dissatisfaction and AB towards one’s own body, it would be valuable to explore whether similar patterns of AB are present in young people with overweight and obesity. Understanding these dynamics could help to develop specific therapeutic interventions for this group and prevent body dissatisfaction. In BED, where food-related AB is pronounced, (cognitive) training opportunities to increase inhibitory control and reducing impulsivity and AB may be helpful in the treatment of young people with overweight, obesity, BED and LOC symptoms. Note that existing trainings, such as attention bias modification training have currently failed to show strong treatment effects in adults with BED symptoms [136]. Yet, improving such as trainings and adapting them to make them suitable and attractive for children and adolescents may be a promising future research avenue.

Given the inconsistent findings to date, further research and the improvement or standardization of methods in studying AB in individuals with EDs and overweight/obesity are essential to develop targeted interventions and preventive strategies for young people.

## Conclusion

In conclusion, while this review underscores the importance of ABs for food and body cues in young people with EDs and overweight/obesity, the current evidence base is limited and fragmented, with methodological inconsistencies that make results difficult to interpret. Furthermore, the understanding of ABs in the broader spectrum of EDs (e.g., BN, BED) remains limited as the focus is predominantly on specific disorders such as AN. Future research should focus on reliable and contextually relevant methods to better capture attentional processes and include more diverse, underrepresented groups, considering developmental and cultural differences. Addressing these limitations will improve our understanding of ABs and facilitate the development of targeted, effective interventions for young people across the ED spectrum and for young people with overweight or obesity.

## Supplementary Information

Below is the link to the electronic supplementary material.

Supplementary Material 1

## Data Availability

Data sharing is not applicable to this article as no datasets were generated or analyzed during the current study.

## Abbreviations

AB: Attentional Bias
AN: Anorexia Nervosa
AN-BP: Anorexia Nervosa Binge-Eating/ Purge Type
AN-R: Anorexia Nervosa Restricting Type
AOI: Area Of Interest
ARDPEI: Attentional Response to Distal versus Proximal Emotional Information task
BED: Binge Eating Disorder
BN: Bulimia Nervosa
BMI: Body Mass Index
CG: Comparison Group
DEBS: Disordered Eating Behaviors
DSM: Diagnostic and Statistical Manual of Mental Disorders
EEG: Electroencephalography
ED: Eating Disorder
ERPs: Event-related Potentials
ET: Eye-Tracking
HC: Healthy Controls
LOC: Loss of Control
LPP: Late Positive Potential
PROSPERO: Prospective Register of Systematic Reviews
ROI: Region of Interest
RT: Reaction Time
WHO: World Health Organization
