# Supplementary material for: Food and body-related attentional biases in children and adolescents with eating disorder symptoms, overweight and obesity: a systematic review
Source: J Eat Disord. 2025 Dec 10;14:9. doi: 10.1186/s40337-025-01459-9 (PMC12781851; doi:10.1186/s40337-025-01459-9)
Supplement: Supplementary file 1 — Supplementary Material 1 [file 40337_2025_1459_MOESM1_ESM.docx]

**Additional file 1**

*Search Terms*

PsychInfo:

("attention* bias“ OR "visual probe“ OR "dot probe“ OR
"visual search“ OR "stroop" OR "eye movements“ OR "EEG" OR
"electroencephal*“OR "ERP*" OR "event-related potentials") AND
("bodies*" OR "body*" OR "shape*" OR "food*") AND
(“adolescent*" OR "child*" OR "girl*" OR “boy*” OR "youth*" OR
"teenage*" OR "underage*" OR "school*")

PubMed:

**((attention* bias[Title/Abstract] OR visual probe[Title/Abstract] OR dot probe[Title/Abstract] OR visual search[Title/Abstract] OR stroop[Title/Abstract] OR eye movements[Title/Abstract] OR EEG[Title/Abstract] OR electroencephal*[Title/Abstract] OR ERP*[Title/Abstract] OR event-related potentials[Title/Abstract]) AND (bodies* [Title/Abstract] OR body* [Title/Abstract] OR shape* [Title/Abstract] OR food* [Title/Abstract]) AND (adolescent* [Title/Abstract] OR child* [Title/Abstract] OR girl* [Title/Abstract] OR boy* [Title/Abstract]OR youth* [Title/Abstract] OR teenage* [Title/Abstract] OR school* [Title/Abstract]) AND ((german[Language]) OR english[Language])) Filters: Clinical Study, Clinical Trial, Comparative Study, Meta-Analysis, Randomized Controlled Trial, Review, Systematic Review**

Scopus:

TITLE-ABS (("attention* bias“) OR ("visual probe“) OR ("dot probe“) OR ("visual search“) OR "stroop" OR ("eye movements“) OR "EEG" OR "electroencephal*“OR "ERP*" OR ("event-related potentials")) AND TITLE-ABS ("bodies*" OR "body*" OR "shape*" OR "food*") AND TITLE-ABS(„adolescent*" OR "child*" OR "girl*" OR “boy*” OR "youth*" OR "teenage*" OR "underage*" OR "school*")

**Additional file 2**

*Study quality rating for studies investigating AB to food-related stimuli*

| Author (s) (year) | Study quality |
| --- | --- |
| Biehl et al. [70] | Fair |
| Hofmann et al. [71] | Good |
| Kösling et al. [72] | Fair |
| Woltering et al. [74] | Good |
| Braet & Crombez [77] | Fair |
| Fearnbach et al. [73] | Fair |
| Rojo-Bofill et al. [78] | Fair |
| Soetens & Braet [80] | Fair |
| Vervoort et al. [79] | Fair |
| Akcay et al. [81] | Poor |
| Folkvord et al. [82] | Fair |
| Werthmann et al. [41] | Good |
|  |  |
| Novosel et al. [84] | Fair |
| Stonawski et al. [85] | Good |
| Jonker et al. [83] | Fair |
| Horndasch et al. [86] | Poor |
| Werthmann et al. [52] | Good |
| Schmidt et al. [87] | Good |
| Shank et al. [90] | Fair |
| Van Malderen et al. [91] | Fair |
| Van Malderen et al. [92] | Fair |

*Study quality rating for studies investigating AB to body-related stimuli*

| Author (s) (year) | Study Quality |
| --- | --- |
| Bauer et al. [95] | Good |
| Pinhas et al. [96] | Fair/Poor |
| Sfärlea et al. [97] | Good |
| Svaldi et al. [98] | Fair |
| Horndasch et al. [99] | Fair |
| Horndasch et al. [100] | Poor |
| Romero Frausto et al. [101] | Good |
| Bauer et al. [93] | Good |
| Horndasch et al. [94] | Fair |
